# Supplementary figures and images for: Cutaneous expression of growth-associated protein 43 is not a compelling marker for human nerve regeneration in carpal tunnel syndrome
Source: PLoS One. 2022 Nov 16;17(11):e0277133. doi: 10.1371/journal.pone.0277133 (PMC9668135; doi:10.1371/journal.pone.0277133)

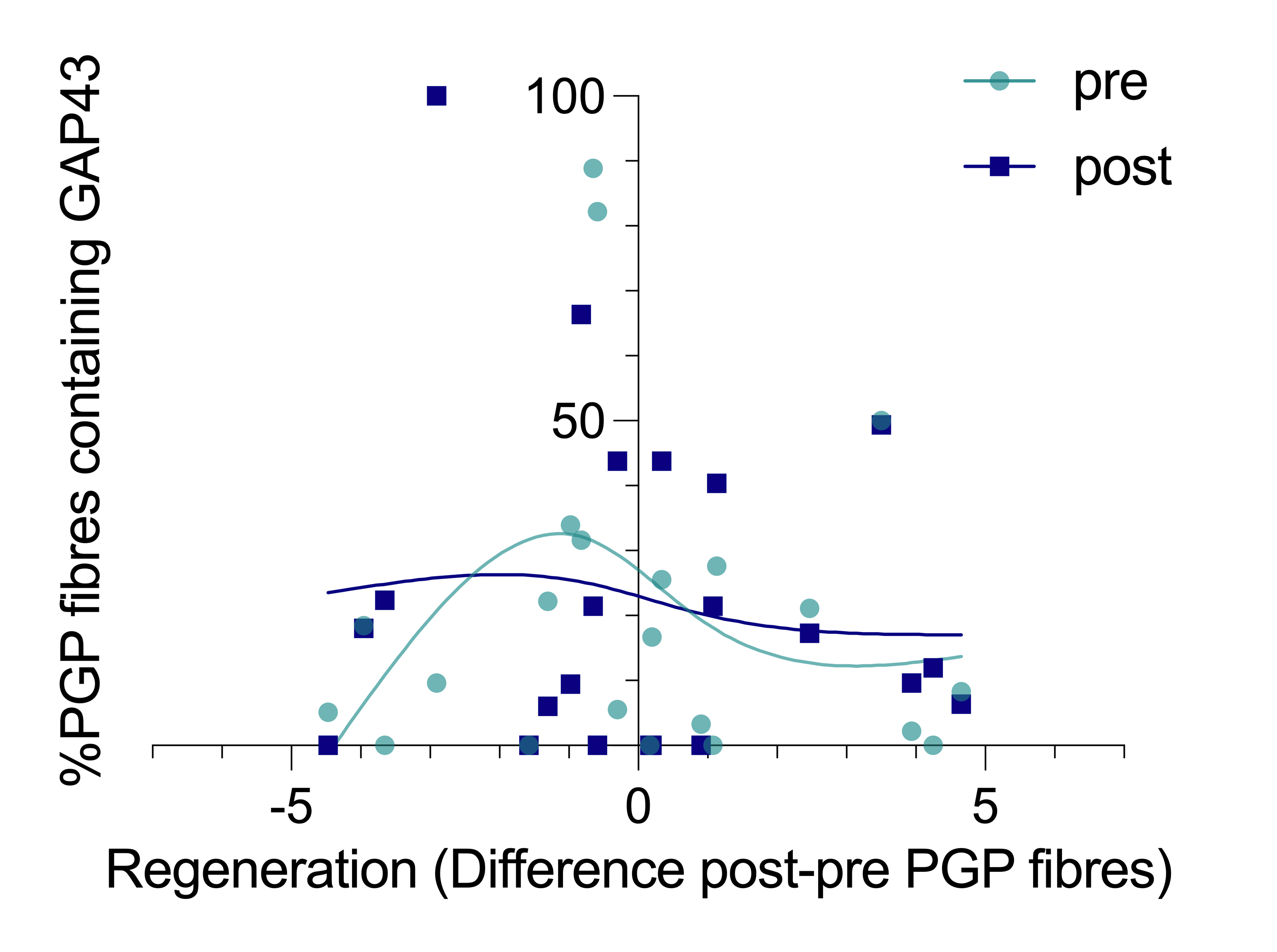

Supplement: S1 Fig — Graph shows pre surgical (green) and post-surgical (blue) PGP+ IENF containing GAP-43 with cubic spline curves showing no association. (TIFF) [file pone.0277133.s001.tiff]
