# Supplementary material for: Cutaneous expression of growth-associated protein 43 is not a compelling marker for human nerve regeneration in carpal tunnel syndrome
Source: PLoS One. 2022 Nov 16;17(11):e0277133. doi: 10.1371/journal.pone.0277133 (PMC9668135; doi:10.1371/journal.pone.0277133)
Supplement: S1 Table — Data are presented as median [interquartile range]. (DOCX) [file pone.0277133.s002.docx]

**S1 Table: Median nerve neurophysiology data of healthy participants and patients with CTS pre- and post-surgery.** Data are presented as median [interquartile range].

|  | CTS patients | | Healthy controls |
| --- | --- | --- | --- |
|  | pre | post |  |
| SNAP (μV) | 6.8 [9.8] | 7.2 [8.2] | 12.4 [16.1] |
| Sensory NCV (m/s) | 36.1 [8.9] | 42.0 [7.6] | 49.2 [10.7] |
| Distal motor latency (ms) | 5.3 [2.2] | 2.3 [0.3] | 3.5 [0.9] |
| CMAP (mV) | 4.8 [4.4] | 6.3 [1.1] | 8.9 [4.2] |
| Delay ulnar-median | 1.6 [1.4] | 0.8 [0.4] | 0.2 [0.4] |

SNAP: sensory nerve action potential; NCV: nerve conduction velocity; CMAP: compound motor action potential; delay ulnar-median: delay between the motor potentials for dorsal interossei (ulnar nerve) and lumbricals (median nerve) measured over an 8cm wrist segment.
