# Supplementary material for: Cutaneous expression of growth-associated protein 43 is not a compelling marker for human nerve regeneration in carpal tunnel syndrome
Source: PLoS One. 2022 Nov 16;17(11):e0277133. doi: 10.1371/journal.pone.0277133 (PMC9668135; doi:10.1371/journal.pone.0277133)
Supplement: S2 Table — Table shows p-values. No significant correlations were identified. (DOCX) [file pone.0277133.s003.docx]

**S2 Table: Spearman correlations between pre and post-operative cutaneous GAP-43 expression (histological analysis) and pain characteristics according to the Neuropathic Pain Symptom Inventory.** Table shows p-values. No significant correlations were identified.

|  | Burning | Deep | Paroxysmal | Evoked | Paraesthesia | Total score |
| --- | --- | --- | --- | --- | --- | --- |
| **Pre-surgery** |  |  |  |  |  |  |
| GAP-43 | 0.921 | 0.139 | 0.739 | 0.375 | 0.222 | 0.326 |
| % of PGP+ IENF containing GAP-43 | 0.776 | 0.117 | 0.377 | 0.282 | 0.061 | 0.088 |
| **Post-surgery** |  |  |  |  |  |  |
| GAP-43 | 0.823 | 0.851 | 0.692 | 0.678 | 0.191 | 0.573 |
| % of PGP+ IENF containing GAP-43 | 0.953 | 0.257 | 0.931 | 0.916 | 0.094 | 0.433 |

IENF: intraepidermal nerve fibres
