# Supplementary material for: Cutaneous expression of growth-associated protein 43 is not a compelling marker for human nerve regeneration in carpal tunnel syndrome
Source: PLoS One. 2022 Nov 16;17(11):e0277133. doi: 10.1371/journal.pone.0277133 (PMC9668135; doi:10.1371/journal.pone.0277133)
Supplement: Attachment — Submitted filename: 05.14.22 Plos one review .docx [file pone.0277133.s004.docx]

Manuscript number: PONE-D-22-11884

Title: **Cutaneous expression of Growth-Associated Protein 43 is not a compelling marker for human nerve regeneration after chronic focal nerve injury**.

Overview and general recommendation:

GAP43 is a well-known marker for neuronal development/growth in embryo and regeneration after neuronal injury in adult rodent animal. Capral tunnel syndrome (CTS) is a typical model both for human sensory nerve degeneration due to nerve compression and regeneration after surgery decompression. The strength of the paper is that Carrol et al choose the optimized model to investigate the difference of GAP43+ immune-positive cutaneous nerve fibers before and after surgery and whether GAP43 expression is related to after surgery regeneration; The authors provided the meaningful and realistic results of GAP43+ fiber regeneration in chronic nerve injury of CTS in patients. This article is acceptable for publication after the. following points are addressed.

The major concerns:

1. Title: As this study only investigates the carpal tunnel syndrome patient’s index finger skin sample with average symptom duration 37.6 months, which belongs to chronic neuronal injury, it should be cautioned to expand the conclusion to all chronic focal nerve injury which may involve different mechanisms. I suggest that the title should be specified to carpal tunnel syndrome only.
2. The GAP43 immunofluorescence staining image: The study measures and expresses the GAP43+ fiber in a reasonable scientific way (Fig 1 and 2) with matched statistics calculation. I strongly suggest the authors provide data from PGP9.5 and GAP43 staining to show the audience that both fibers are effectively detected.
