# Supplementary material for: Cutaneous expression of growth-associated protein 43 is not a compelling marker for human nerve regeneration in carpal tunnel syndrome
Source: PLoS One. 2022 Nov 16;17(11):e0277133. doi: 10.1371/journal.pone.0277133 (PMC9668135; doi:10.1371/journal.pone.0277133)
Supplement: Attachment — Submitted filename: 08.15.22 2nd review final 2.docx [file pone.0277133.s005.docx]

**08.15.22 Resubmission review**

1. **Title**. I really appreciate that the author changed the title to make it specific to carpal tunnel syndrome.
2. **The GAP43 immunofluorescence staining lacks clarity**. In figure 2, the PGP9.5 staining appears correct, however the GAP43 staining presents a problem. The GAP43 Cy3 color completely overlaps with PGP9.5 (AlexaFluor-488) and fails to show any difference between the two nociceptors. It is true that most GAP43+ fibers are usually positive for PGP9.5. However, they are different nociceptors, derived from different types of DRG neuron, thus they have their own unique morphology, and should not completely overlap in immunofluorescence staining. Another weakness of this figure: the nerve bundle traveling inside the dermis is well presented, but the terminals located inside the epidermis, which is the important part, are not well stained and it is difficult to discern the two types of nociceptors. Overall, figure 2 does not correctly demonstrate the difference of these two types of nociceptors. Current, it is not proper to accept it for publication.
