# Supplementary material for: Cutaneous expression of growth-associated protein 43 is not a compelling marker for human nerve regeneration in carpal tunnel syndrome
Source: PLoS One. 2022 Nov 16;17(11):e0277133. doi: 10.1371/journal.pone.0277133 (PMC9668135; doi:10.1371/journal.pone.0277133)
Supplement: Attachment — Submitted filename: Response to reviewers 2.docx [file pone.0277133.s006.docx]

**Point by point reply**

Reviewer 1: The GAP43 immunofluorescence staining image needs minor revise. In figure 2, the PGP9.5 staining appears correct, however the GAP43 staining presents minor problem. Most of the GAP43 Cy3 color overlaps with PGP9.5 (AlexaFluor-488) and fails to show the difference between the two nociceptors. It is true that most GAP43+ fibers are usually positive for PGP9.5, even they might be different nociceptors, derived from different

types of DRG neuron, thus they should have their own unique morphology. The overlapped immunofluorescence staining is a weakness of this image.

Another weakness of this figure is the nerve bundle traveling inside the dermis is well presented, however, the axon terminals located inside the epidermis which is the most important part to distinguish the different type of nociceptor are not well stained. Therefore, it is difficult to discern the two types of nociceptors. Overall, considering the weakness of figure 2, I would suggest the author provide additional PGP9.5 and GAP43 double staining image with the emphasis on the fiber located within epidermis as a minor revise.

We would like to thank the reviewer for this comment. We have now taken new images of the double staining of PGP and GAP-43 and revised our figure accordingly.

However, we are somewhat puzzled by the comment. PGP9.5 stains all nerve fibres, it is a pan-axonal stain. There are therefore no nerve fibres that are GAP-43 positive but PGP9.5 negative. As such GAP-43 does not stain a different subset of nociceptors than PGP9.5 would, but rather GAP-43+ neurons are a subset within PGP+ nerve fibres. We agree though that in the image we provided, most PGP+ fibres were also GAP-43 positive. That is because we selectively chose a section of a patient with overlap to show the staining quality.

We have now revised our figure to include a representative image which shows some PGP+ intraepidermal nerve fibres with and some without GAP-43 labelling. We have highlighted these with arrows and arrow heads respectively.

We have now also delineated the dermal/epidermal border to help the readers distinguish between dermal and intraepidermal nerve fibres, as in fact our previous image contained many intraepidermal nerve fibres. Please note that the morphology of finger skin is quite distinct (e.g., many papillary indentations) from the leg. This may have led to the misinterpretation of the images to only contain dermal fibres. We hope that these revisions and the new images will help showcase the quality of our staining as well as the double labelling better.
